# Pulmonary Embolism as a Potential Trigger for Takotsubo Cardiomyopathy

**DOI:** 10.7759/cureus.62342

**Published:** 2024-06-13

**Authors:** Hakob Harutyunyan, Supraja Achuthanandan, Vahagn Tamazyan, Aung Oo, Aleksan Khachatryan, Ashot Batikyan, Meghana Shetty, Vijay Shetty

**Affiliations:** 1 Department of Internal Medicine, Maimonides Medical Center, New York, USA; 2 Department of Cardiology, Maimonides Medical Center, New York, USA; 3 Department of Medicine, Maimonides Medical Center, New York, USA; 4 Department of Internal Medicine, University of Maryland Medical Center Midtown Campus, Baltimore, USA; 5 Department of Internal Medicine, North Central Bronx Hospital, New York, USA; 6 Department of Medicine, K S Hegde Medical Academy, Mangalore, IND

**Keywords:** broken heart syndrome, left ventricle apical ballooning, catecholamine surge, takotsubo cardiomyopathy, pulmonary embolism

## Abstract

We present a case of an 83-year-old female who presented to the emergency department because of poor oral intake and dizziness. Initial assessment revealed a diagnosis of pulmonary embolism (PE). However, further investigation revealed coexisting takotsubo cardiomyopathy (TCM), a rare but critical finding. This case highlights a possible causative connection between clinically non-significant PE and TCM. Additionally, it emphasizes the diagnostic challenges posed by atypical symptoms and unremarkable medical history, which can lead to delayed diagnosis in such cases.

## Introduction

Pulmonary embolism (PE) and takotsubo cardiomyopathy (TCM) are distinct cardiovascular conditions with significant clinical role. PE is a severe and often life-threatening condition that arises from the obstruction of the pulmonary arteries, typically due to a thrombus from the deep veins of the legs or pelvis [1]. This obstruction impedes blood flow, increasing strain on the right side of the heart and leading to right heart failure [1]. Its presentation can vary widely, from asymptomatic to sudden death, with typical clinical manifestations such as shortness of breath, chest pain, and hemoptysis [1]. Early diagnosis and prompt treatment can be crucial, as the condition carries a high mortality risk, particularly in the first few hours following the embolic event [1].

On the other hand, TCM is represented by transient left ventricular (LV) dysfunction in the absence of significant coronary artery obstruction [2]. It is often precipitated by acute emotional or physical stress and imitates acute coronary syndrome in its presentation [3,4]. The exact etiology remains unclear, but a surge in catecholamines leading to myocardial stunning is widely believed to be a key component in its pathogenesis [5]. Despite its dramatic presentation, TCM is often reversible, and most patients demonstrate complete recovery within weeks [3]. Patients with TCM experience an annual all-cause mortality rate of 5.6% and an annual major adverse cardiac and cerebrovascular event rate of 9.9% [2]. Both PE and TCM can present with similar clinical manifestations, including chest pain and dyspnea [6]. However, their simultaneous occurrence is a diagnostic dilemma and a potential clinical challenge due to the additive strain on the heart. Understanding the nuances of each condition and the implications of their coexistence is essential for optimal patient care.

In the context of these complex connections, we demonstrate a unique case of a female patient with non-traditional symptoms who was concurrently diagnosed with both PE and TCM.

## Case presentation

An 83-year-old female presented to the emergency room with complaints of poor oral intake and dizziness. The patient denied experiencing chest pain, shortness of breath, headache, abdominal pain, or diarrhea during the initial assessment. Notably, she reported pain in the coccyx area attributed to a previous coccygeal fracture. The patient also mentioned chronic left lower extremity swelling and tenderness. Additionally, she had a history of a blood clot in her leg diagnosed about five years ago. Despite these medical issues, the patient was able to ambulate with the assistance of a walker. The patient reported no recent emotional distress, travel, or personal history of cancer. Her medical history was significant for hypertension, hyperlipidemia, deep vein thrombosis not on anticoagulation, and bilateral lower extremity stents placed approximately five years ago. On physical examination, the patient was found to have mild non-pitting edema and tenderness of the left lower extremity. The cardiopulmonary examination was otherwise unremarkable. 

**Figure 1:**
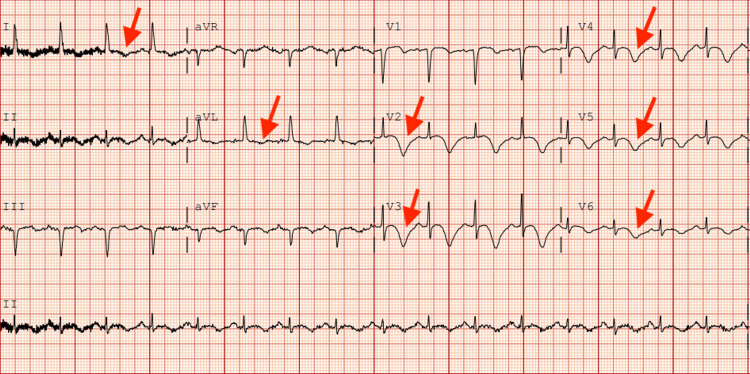
Initial electrocardiogram on admission Red arrows depict T wave inversions in leads I, aVL, V2-V6.

Vital signs were as follows: temperature of 37°C, heart rate of 102 beats per minute, blood pressure of 101/56 mmHg, respiratory rate of 20 breaths per minute, and oxygen saturation of 97% on room air. The laboratory findings on admission revealed a serum troponin level of 0.03 ng/mL (0-0.02 ng/mL) which uptrended to 2.13 ng/mL, and B-type natriuretic peptide (BNP) 889 pg/mL (<100 pg/mL), otherwise unremarkable. Initial electrocardiogram (ECG) demonstrated diffuse T inversions in leads I, aVL, V2-V6 (Figure 1).

**Figure 2:**
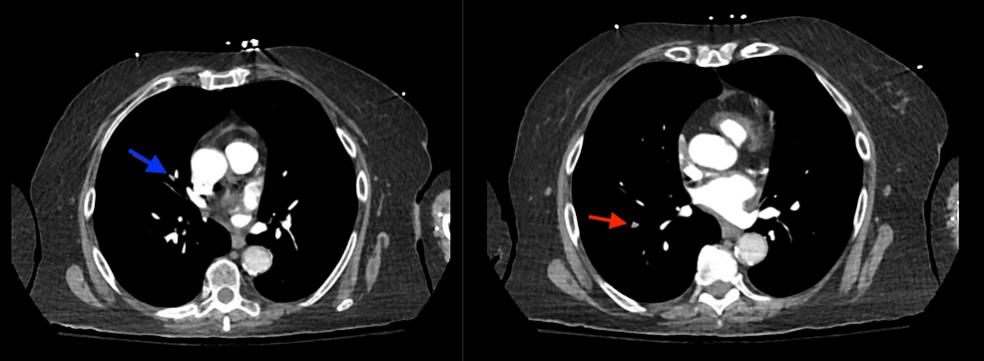
Computed tomography angiography of the chest A filling defect is noted in the bifurcation of the proximal segmental right middle lobe pulmonary artery and extends into the lateral subsegmental branch (blue arrow). A focal filling defect causing abrupt cutoff of the right lower lobe lateral basal subsegmental branch is also noted (red arrow).

Computed tomography angiography (CTA) of the chest demonstrated right middle and lower lobe subsegmental PE (Figure 2).

**Figure 3:**
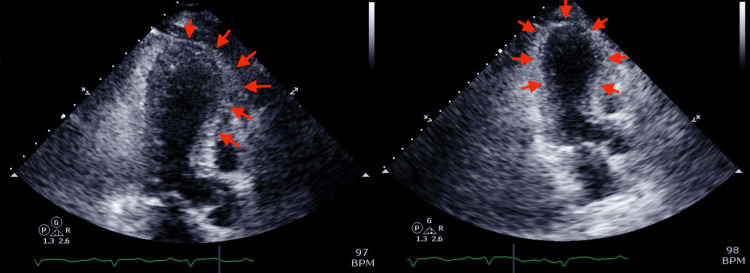
Echocardiogram on admission Left ventricular apical ballooning (depicted by red arrows) is suggestive of takotsubo cardiomyopathy.

Initial echocardiogram revealed left ventricular ejection fraction (LVEF) of 36-40% with regional LV systolic wall motion abnormality suggestive of TCM. Right ventricular (RV) systolic function was normal (Figure 3).

**Figure 4:**
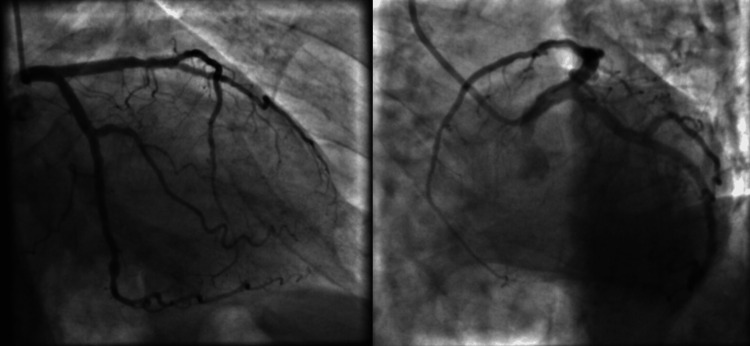
Coronary angiography of the left coronary artery branches There are no hemodynamically significant coronary artery lesions.

The patient's coronary angiography did not show any hemodynamically significant abnormalities, which further supported the diagnosis of TCM (Figure 4).

The patient was promptly started on therapeutic anticoagulation with heparin and admitted to the intensive care unit for close monitoring. Subsequently, heparin was switched to apixaban, and the patient was started on guideline-directed medical therapy for heart failure.

**Figure 5:**
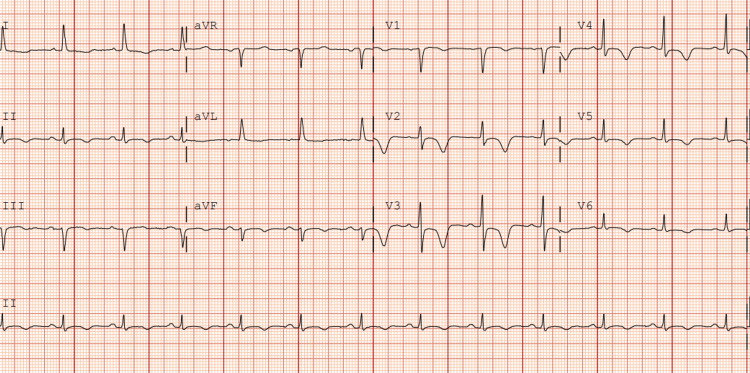
Electrocardiogram on discharge No significant changes compared with the initial ECG.

**Figure 6:**
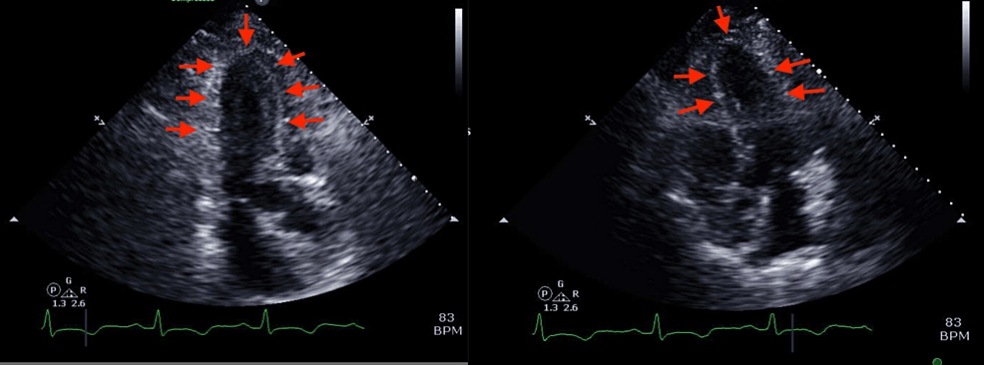
A repeated echocardiogram was performed five days later. Red arrows depict resolved left ventricular apical ballooning seen in initial imaging.

During the hospital course, the patient remained clinically stable. Five days later, repeated ECG did not show any significant changes (Figure 5) and echocardiography revealed normalized LV function with EF of 56-60% (Figure 6).

The patient was discharged with appropriate medications and scheduled for regular outpatient follow-up.

## Discussion

The simultaneous occurrence of PE and TCM presents a unique intersection that can challenge even experienced clinicians. While both conditions can manifest with similar symptoms, such as dyspnea, chest pain, and elevated cardiac biomarkers, their coexistence is rare, and the mechanism underlying this simultaneous presentation is still not entirely clear. PE, arising from a thromboembolic event, can significantly strain the right side of the heart [7]. While there wasn't any evidence of right heart strain in our case, increased pulmonary vascular resistance could elevate ventricular afterload, potentially precipitating RV dysfunction and eventual failure [7].

TCM, often termed stress cardiomyopathy, is believed to result from an excessive catecholamine surge, which causes myocardial stunning [4]. This could lead to the observed LV wall motion abnormalities typical for TCM [5]. Given the described pathophysiological mechanisms, elevated stress on the heart can lead to a surge in catecholamine levels, potentially precipitating TCM in cases of PE [5].

Distinguishing between PE and TCM based only on clinical presentation can be challenging due to symptom overlap. Yet, the differential diagnosis becomes even more challenging when they coexist. In our case, the patient's atypical presentation and absence of typical symptoms like chest pain or shortness of breath compounded the diagnostic puzzle. This highlights the importance of maintaining a broad differential diagnosis and the role of advanced diagnostic tools like point-of-care ultrasound in guiding clinical decisions. Notably, when faced with a patient presenting with TCM without an apparent causative history, the possibility of PE as an underlying etiology, regardless of its severity, should remain on the differential [8].

In contrast, patients diagnosed with PE with reduced EF might benefit from further evaluation for TCM as a potential, reversible etiology of their cardiac dysfunction [9]. This case is a potential reminder that the heart's response to acute stressors is complex. While PE and TCM each have distinct pathophysiological mechanisms, they may intertwine in ways that aren't immediately apparent, thereby shaping the clinical picture. Clinicians should be vigilant and consider that seemingly unrelated cardiac events might be interconnected. Such awareness can guide appropriate investigations, prompt diagnosis, and timely therapeutic interventions, crucial for optimizing patient outcomes.

## Conclusions

The coexistence of PE and TCM highlights cardiovascular pathologies' complexity and interrelated nature. This case illustrates clinicians' challenges in diagnosing and managing such complications, especially when presented with atypical manifestations. It emphasizes maintaining a comprehensive diagnostic approach, especially in cases where symptoms overlap or appear contradictory. The potential relationship between the stress of PE and the catecholamine surge in TCM offers a compelling avenue for further research. Clinicians should remain vigilant, recognizing the cardiovascular system's diverse and sometimes unexpected reactions to acute stressors. By conducting thorough assessments and developing a comprehensive understanding of the potential interactions among different conditions, we can work towards achieving the most favorable outcomes for our patients.
